# Tracing the impact of CO_2_ on the electrochemical and charge–discharge behavior for Al–Mg alloy in KOH and LiOH electrolytes for battery applications

**DOI:** 10.1038/s41598-024-57638-2

**Published:** 2024-04-02

**Authors:** Abdelrahman El-sayed, Mohamed Abdelsamie, Mahmoud Elrouby

**Affiliations:** 1 Chemistry Department, Faculty of Science, Sohag University, Sohâg, 82524 Egypt; 2 Faculty of Science, King Salman International University, Ras Sedr, Sinai 46612 Egypt

**Keywords:** Al–Mg alloy, Electrochemical performance, Li-ions, Corrosion resistance, CO_2_, Electrochemistry, Energy, Physical chemistry

## Abstract

For the first time, it has been found that the electrochemical performance of the Al–Mg alloy as an anode in alkaline batteries has been markedly enhanced in the presence of CO_2_ and LiOH as an electrolyte. This work compares the electrochemical performance of an Al–Mg alloy used as an anode in Al-air batteries in KOH and LiOH solutions, both with and without CO_2_. Potentiodynamic polarization (Tafel), charging-discharging (galvanostatic) experiments, and electrochemical impedance spectroscopy (EIS) are used. X-ray diffraction spectroscopy (XRD) and a scanning electron microscope (SEM) outfitted with an energetic-dispersive X-ray spectroscope (EDX) were utilized for the investigation of the products on the corroded surface of the electrode. Findings revealed that the examined electrode’s density of corrosion current (*i*_corr._) density in pure LiOH is significantly lower than in pure KOH (1 M). Nevertheless, in the two CO_2_-containing solutions investigated, *i*_corr._ significantly decreased. The corrosion rate of the examined alloy in the two studied basic solutions with and without CO_2_ drops in the following order: KOH > LiOH > KOH + CO_2_ > LiOH + CO_2_. The obtained results from galvanostatic charge–discharge measurements showed excellent performance of the battery in both LiOH and KOH containing CO_2_. The electrochemical findings and the XRD, SEM, and EDX results illustrations are in good accordance.

## Introduction

Battery anodes made of aluminum are promising because of their intrinsic characteristics, such as high volumetric energy capacities of 8046 mA h cm^−3^, high gravimetric energy capacities of 2980 mA h g^−1^, and cathode voltages of − 2.31, − 2.30, − 1.68 V versus SHE in acidic, neutral, and basic electrolytes, respectively^1–4^. The batteries of aluminum-air (Al-air) are considered a wonderful technology for storing energy due to their abundant reserves, low cost, and lightweight, making them attractive for stationary power stations and electric vehicles^5–8^. It is essential to note that improving the Al anode for Al-air batteries is exceedingly lethargic, mostly due to two fundamental difficulties. At first, it is assumed that the internal resistance of the protection film adhering to the Al electrode causes the high overpotential^9^. Second, anode self-corrosion, considered the greatest challenge, considerably reduces the aluminum anode’s coulombic effectiveness. Alkaline solutions (KOH or NaOH) are frequently utilized in Al-air batteries because they give a high operating overpotential and a high discharging current. They may also break down the Al anode’s passivated layer, enabling continued discharge. Indeed, Al anode self-corrosion in alkaline electrolytes generates considerable self-discharge as the consequence of low anode consumption and delays the Al batteries commercialization^10–12^. Lithium-ion (Li-ion) batteries have become the most significant devices for storing energy in recent decades due to their high energy density, high output voltages, high durability, low emission, non-toxic, and environmentally safe characteristics^13^. Al anodes are fantastic anode materials for batteries of lithium-ion^14^. Nevertheless, the pristine aluminum anode in LIBs (Li-ion batteries) has significant drawbacks, such as a short cycle life and a high irreversible capacity loss. Due to the tremendous volume expansion during lithiation and de-lithiation, the loss of electrical contact between individual particles and the current collector causes a rapid decrease in capacity^15^.

To address the aforementioned challenges, the most popular and effective method is called alloying, which requires combining aluminum with a specified element. Many alloying elements, particularly Sn, In, Ga, Mg, Hg, Mn, Pb, Zn, Bi, and Sb, were examined during the last several years^16^. Mg is typically incorporated as one of the doped metals in hybrid material Al alloy anodes^1,17^. Adding Mg to Al is suggested to enhance its susceptibility towards impurities because Mg produces compounds with metals like Si that decrease the number of cathodic sites^18^. Gao^19^ also found that Mg addition reduces the Al anode’s dissolution rate. Ren et al.^20^ proved that Mg could lower pure Al’s surface energy and limit self-corrosion’s speed. Liang et al*.*^21^ examined how the magnesium content of the Al–Mg–Sn–Ga–Pb quinary alloy anode affected its microstructure and electrochemical characteristics. Li et al.^22^ employed polarization plots and electrochemical impedance spectroscopy to demonstrate that Mg inclusion is vital to improving the discharge effectiveness of an Al-based anode. Secondly, Li-ions are used as an electrolyte source to develop the battery. Gallagher et al.^23^ found that LiOH is viable for enhanced Li–O_2_ batteries that can function in humid conditions. Current Li–O_2_ batteries must remove moisture from the air down to a few ppm, increasing cost and energy density. Moreover, Zhang et al.^24^ established the high energy efficiency and rate capabilities of cycling Li–O_2_ batteries based on LiOH. Also, the ionic conductivity of Li^+^ ions was demonstrated by Wang et al*.*^25^ to be around 0.1 m Siemens/cm at ambient temperature. Wang et al*.*^26^ outlined the most current developments in anode materials based on aluminum and the associated lithium storage system for lithium-ion batteries. The influence of CO_2_ has been studied on many metals and has a corrosive character in neutral and acidic solutions^27,28^. The literature review found that there has never been any research on aluminum or its alloys in a basic media containing carbon dioxide^29–31^, except for our published paper at this point^32^. This published paper studied the effect of Sn and Zn alloying with Al on its electrochemical performance in KOH solution containing CO_2_ for Al-air batteries application. It is found that CO_2_ enhanced the electrochemical performance of Al more than that of its alloys, and the mechanism of Al reactions was suggested as the following:

In the absence of CO_2_:

1 $$ {\text{5Al }} + {\text{ 15OH}}^{ - } \to {\text{3Al}}\left( {{\text{OH}}} \right)_{{3}} + {\text{ Al}}_{{2}} {\text{O}}_{{3}} + {\text{ 3H}}_{{2}} {\text{O}} $$

In the presence of CO_2_:

2 $$ {\text{2Al }} + {\text{ 3CO}}_{{2}} + {\text{ 6OH}}^{ - } \to {\text{Al}}_{{2}} \left( {{\text{CO}}_{{3}} } \right)_{{3}} + {\text{ 3H}}_{{2}} {\text{O}} $$

This exhibits that the formation of insoluble carbonate of Al on its surface in the presence of CO_2_ plays an important role in suppressing the corrosion process more than in its absence.

Until now, to the best of our knowledge, CO_2_ gas emissions have dangerous problems of global warming^33^. It is noticed that greenhouse gases (mostly CO_2_), which are released while using fossil fuels, cause a thorny problem of threatening human survival^34^. Many types of renewable energy, such as solar, tidal, and wind, can be utilized instead of fossil fuels. However, the mentioned energy sources are unstable, and the storage cost is high^35^. Recently, very rare work has been done on the batteries based on CO_2_ have been developed to diminish the dependence on traditional fossil fuels and establish CO_2_^29^. This study will compare the performance of Al-air batteries in KOH and LiOH electrolytes in the presence and absence of CO_2_ using a modified anode (Al–Mg alloy). This can be accomplished by identifying the corrosion characteristics and charge–discharge properties to understand the implications of Li^+^ ions and CO_2_. In order to imitate the actual working temperature of the Al-air battery system, tests are also conducted up to 50 °C^36^. As a consequence, stimulating and suppressing the corrosion impact of magnesium inclusion and the presence of carbon dioxide have been solidified through an extensive electrochemical investigation. This research can be used as a starting point for developing multicomponent Al alloy anodes in the presence of CO_2_ for alkaline batteries.

## Experimental

### Materials and chemicals

An electrolyte solution of 1 Molar of potassium hydroxide and lithium hydroxide was made by using a measuring flask to dissolve a predetermined amount of electrolyte solute in double distilled water. Al and its alloy (Al–Mg) were prepared to utilize commercial Al only and with high-purity Mg in a graphite crucible at 750 °C. Liquid metals are consistently blended by tumult and spit into a tempered steel form (150 mm × 130 mm × 30 mm). After a homogenizing sintering process at 500 °C for 8 h, the as-cast ingots were allowed to cool down within the oven to ambient temperature. Al sheets have been fabricated by first transferring the ingot hot to a thickness of 10 mm at a temperature of 420 °C, then moving the ingot cold to a thickness of 3 mm in multiple passes, then annealing the material at a temperature of 400 °C for 2 h, and finally air cooling it. In the end, aluminum samples were divided into various sizes cubes using silicon carbide sheets with a coarseness range of 400–1500. These cuboids served as electrode materials for the experiment^19^.

It is possible to create and control the saturated solution of CO_2_. By adding CO_2_ to the electrolyte solution over a prolonged period of time—roughly 20 h—at an appropriate flow rate, one may regulate the saturation process. The cylinder holding the pure gas is used to supply the CO_2_. Throughout the bubbling process, the pH of the resultant solution was continually monitored until it reached a stable value of around 9 (pH of the unsaturated solution = 13 for KOH and LiOH). Then the gas is purged over the solution. The pure nitrogen gas is bubbled and purged in the solution to remove the dissolved oxygen gas and any other dissolved gases in the absence of CO_2_, which may be achieved without passing the CO_2_ in the solution.

### Structure characterization

The compositional phases of the fabricated electrode have been estimated by employing a Brucker AXS-D_8_ of a highly developed diffractometer with radiation of 1.54 nm in wavelength in the X-ray scattering lab (XRD). The morphological structure of the electrode surface was examined utilizing a scanning electron microscope (SEM) of the JSM IT 200 type outfitted with an energy-dispersive X-ray spectroscopy analyzer (EDX).

### Set up the electrochemical measurements

All electrochemical tests have been performed in a glass cell made from Pyrex with a three-electrode design. The VersaSTAT4 potentiostat/galvanostat has been utilized. An Al–Mg alloy with a surface area of 0.196 cm^2^ was placed in an Araldite holder and encased in Teflon for insulation. The working electrode was polished with emery paper of increasing grits (800–1200 µm) before being placed in the polarization cell, degreased in pure acetone, and rinsed in flowing bi-distilled water. As a counter electrode, a platinum sheet is employed. As a reference electrode, a saturated silver/silver chloride (Ag/AgCl, sat. KCl) electrode was utilized, with which all potentials were assessed. The working electrode’s surface was electrochemically cleaned by providing a continuous voltage of − 2 V vs. silver/silver chloride electrode for 5 min in each sample electrolyte. The hydrogen bubbles that had collected on the electrode’s surface were then eliminated by unplugging the electrode and shaking it. Consequently, the cathodic and anodic polarization tests, EIS, and charge–discharge were performed.

#### Potentiodynamic polarization (Tafel)

The patterns of Tafel were conducted at varying temperatures (25, 35, 45, 50 °C) with a voltage sweep speed of 1 mV/s, between an applied voltage of − 0.25 and + 0.25 V recognized as a stable open-circuit potential condition (*E*_corr._).

#### EIS measurements

The EIS studies were performed versus open-circuit potential (OCP) using an AC voltage magnitude of 10 m volts and frequency varying from 10 to 0.001 kHz at 25 °C,

### Charging-discharging process

The charging-discharging investigations were accomplished at stable charging and discharging densities of the current (1 m amp. cm^−2^) and voltage limitations of 0 V at varying degrees of temperature in Celsius (25, 35, and 45).

### Determination of the corrosion criteria

For 30 min, the working electrode was immersed in 1 M KOH and LiOH at the OCP for stabilizing. El-Sayed, Ibrahim, et al*.*^37^ reported identifying the crossing point for the extrapolating cathodic and anodized Tafel paths and the densities of *i*_corr._ for the tested electrode could be reliably identified. Freshly prepared solution electrolytes and cleaned electrodes were used for all tests. The Tafel and charge–discharge experiments were carried out at varying temperatures with an ultra-thermostat of model: “Fighter-6000382 SEL ECTA”. At least three times, the same tests were performed with satisfactory repeatability. All the above-mentioned investigations have been reproduced.

## Results and discussion

### Evaluation of the characteristics of the prepared electrode

#### X-ray diffraction spectroscopy (XRD) method

**Figure 1:**
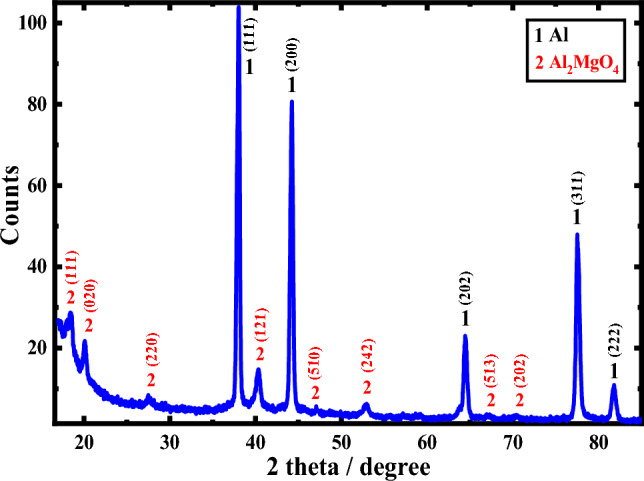
XRD for the pristine surface of the aluminum-magnesium alloy.

Figure 1 displays the X-ray diffraction behavior for the pristine surface of the Al–Mg alloy. The figure exhibits two peaks of aluminum as a high-intensity cubic crystalline. The other three peaks for aluminum also correspond to cubic crystalline but are found with a lower intensity beyond 60° (2θ). The other peaks correspond to the dialuminium magnesium tetroxide (Al_2_MgO_4_) phase of a cubic and orthorhombic crystalline structure. Because of the low ratio of the alloyed magnesium element to aluminum, the peaks of Al_2_MgO_4_ have lower intensities than the other peaks. Noticeably, the system of Al_2_MgO_4_ appeared at three peaks (one peak having high intensity and the other having low). At the same time, the orthorhombic crystalline structure has five peaks (two peaks are high intensity & three are low intensity). In addition, there are no peaks for Mg metal alone without bonding. This means that all the magnesium added during the alloy preparation is linked with the aluminum, which forms the Al_2_MgO_4_ phase.

By applying the well-known Scherer’s formula to the XRD data, the average crystallite size of the alloy constituents was determined based on the analysis of the pattern^38^:

3 $$ D_{hkl} = \frac{K\lambda }{{\beta_{hkl} \cos \theta }} $$

where ${{\text{D}}}_{{\text{hkl}}}$ is the crystallinity size, *λ* the wavelength (Cu *K*_α_ = 1.54 Å), *θ* the reflection angle, *K* the Scherer’s arbitrary value, lies between 0.87 and 1, and ${\beta }_{{\text{hk}}l}$ the full-width of peak half-maximums. From the calculations of the size of the crystal from Scherer’s equation listed in Table 1, it is noted that the size of the grain of the Al_2_MgO_4_ phase is less than the size of the grain of Al in the prepared alloy. This indicates that alloying magnesium with aluminum reduces the size of aluminum by forming the Al_2_MgO_4_ compound. Conversely, the size of the grain of the orthorhombic crystalline system of Al_2_MgO_4_ is greater than that of the cubic crystalline system of the same one. Table 1 summarizes the XRD results for the alloy’s pure surface (Al–Mg). This data includes the 2 (degree) and size of the crystal (nanometers), among other relevant information.

**Table 1:** XRD findings for the non-corroded surface for aluminum-magnesium alloy.

Phase designation	Crystal system	d (A°)	2 (theta)	Card no.	Size of the crystal (nanometers)
Al	Cubic	2.3397	38.444	COD#4313217	23.30
		2.0262	44.688		24.50
		1.4327	65.048		25.03
		1.2219	78.161		25.45
		1.1698	82.369		25.96
Al_2_MgO_4_	Cubic	4.66530	18.328	COD#9005767	14.18
		1.64940	52.682		16.93
		1.36590	67.259		23.72
	Orthorhombic	4.32340	20.052	COD#9010260	20.52
		3.26330	27.307		21.34
		2.28180	40.460		25.26
		1.93930	46.807		27.82
		1.32760	70.392		29.00

#### Scanning electron microscope (SEM) with EDX analysis

**Figure 2:**
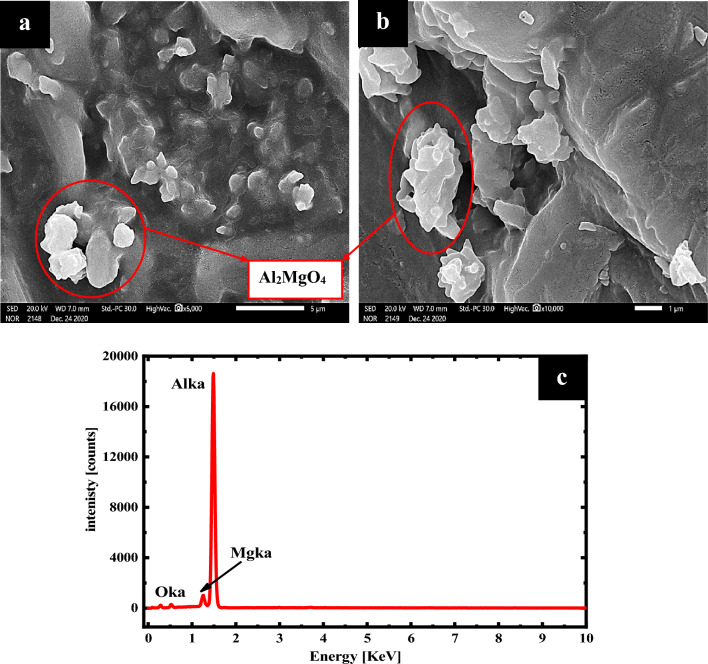
Images obtained from a scanning electron microscope (SEM) at magnifications of 5000 (**a**), 10,000 (**b**), and analyses of EDX charts (**c**) of the pristine surface of an aluminum-magnesium anode.

Figure 2 displays the images that were obtained by the scanning electron microscope and magnified at 5000 (a) and 10,000 (b) times for the Al–Mg alloy that was formed by the fusion process and quenched in accordance with the procedures outlined in the experimental procedure. Due to the generation of dialuminium magnesium tetroxide (Al_2_MgO_4_) in the alloy, the structural morphology of the alloy is smoother than that of commercial Al, as shown in Fig. 2a,b. The picture of SEM for the pristine surface looks to have been scraped by a cutting instrument in a formal manner, as seen by the presence of certain vertical lines throughout the image. The surface seems smooth, with no holes and a few very fine scratches, as seen in the accompanying images (Fig. 2a,b). Moreover, it appears to have a hard surface, confirming the microhardness measurements (measured in Vickers), which were found to be 57.

EDX analysis was conducted to estimate the percent of elements of the synthesized Al–Mg anode, as exhibited in Table 2, and the peaks of EDX in Fig. 2c, as it is clear that aluminum is the major percentage (91.97%) and magnesium is the minor one (3.42%). Oxygen appears at 4.61% in the alloy, the link between aluminum and magnesium in the new phase formed (Al_2_MgO_4_), as shown in the XRD chart (Fig. 1), and achieves the work.

**Table 2:** EDX data of the non-corroded surface of the Al–Mg alloy.

Element	Mass %	Atom %
O K_a_	4.61 ± 0.12	7.52 ± 0.19
Al K_a_	91.97 ± 0.37	88.81 ± 0.35
Mg K_a_	3.42 ± 0.06	3.67 ± 0.07
Total	100.00%	100.00%

### Comparison between the electrochemical performance of Al–Mg electrode in both KOH and LiOH electrolytes with and without CO_2_

#### Potentiodynamic polarization (Tafel region)

**Figure 3:**
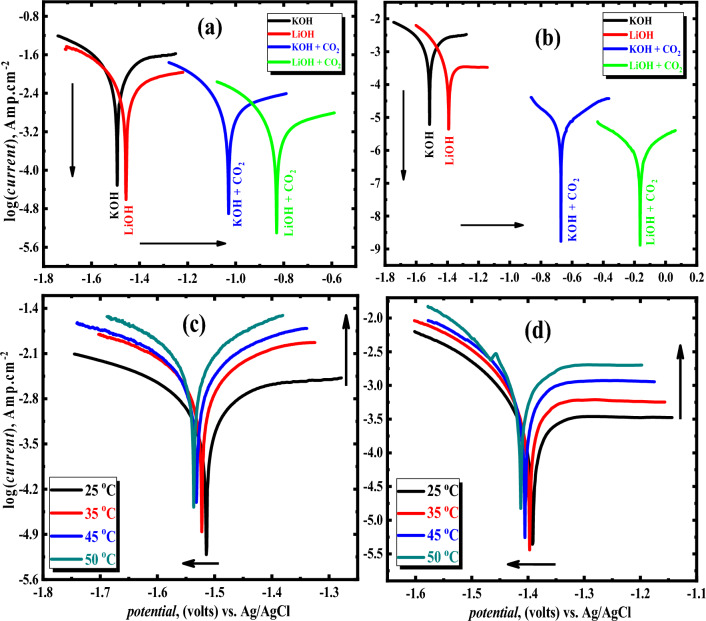
Comparison of the potentiodynamic polarization attitude of the Al (**a**) and Al–Mg (**b**) anodes in 1 M electrolytes of KOH and LiOH with and without saturated CO_2_ at ambient temperature, potentiodynamic polarization plots at different temperatures and a scan rate of 1 m volts/s for Al–Mg in 1 molar of KOH (**b**) and LiOH (**c**).

Figure 3a,b displays potentiodynamic polarization curves at the Tafel region for the Al and its alloy with Mg, respectively in 1 M of each KOH and LiOH solution in the existence and absence of saturated CO_2_ at ambient temperature (25 °C). It can be noted that the *i*_corr._ (listed in Table 3) of the Al electrode is higher than that of Al–Mg alloy in both two electrolytes (KOH and LiOH) in the presence and absence of CO_2_ at ambient temperature. Therefore, the study will be focused on the Al–Mg alloy. According to the results for the Al–Mg electrode (Fig. 3b), both two branches of anodic and cathodic current densities in the LiOH solution are noticeably smaller than those found in the KOH electrolyte. This is due to the quantity of lithium ions seeming to have a significant influence on the amount of aluminum magnesium that corrodes. In addition, the adsorption of Li-ion on the surface of the examined alloy is responsible for the positive movement of the corrosion potential (− 1.391 V vs. Ag/AgCl) and the simultaneous decline in the corrosion rate of the LiOH solution currently faced with the KOH solution (− 1.514 V vs. Ag/AgCl). It has been demonstrated that substituting LiOH for KOH in a solution reduces active sites on the Al–Mg anode surface. As a result, the rate of corrosion is slowed down^39^.

**Table 3:** Parameters of corrosion for Al–Mg alloy and Al metal in 1 M of both KOH and LiOH at various temperatures (25–50 °C), and in the presence of CO_2_ in each of the two investigated solutions were retrieved from Tafel polarization.

Electrode	Electrolyte (1 Molar)	Parameters				
		*I*_corr._ (µ Amp./cm^2^)	− * E*_corr._ (volts vs. Ag/AgCl)	*β*_a_ (m volts/decade)	− *β*_c_ (m volts/decade)	*U*_corr._ (mm/y)
Al	25 °C					
	KOH	2406.17 ± 12	1.49 ± 0.007	74.7 ± 3	48.89 ± 2	26.23 ± 1.3
	KOH + CO_2_	515.48 ± 4	1.03 ± 0.004	74.89 ± 2.5	54.58 ± 2.2	5.62 ± 0.09
	LiOH	1506.05 ± 7	1.45 ± 0.006	104.4 ± 3.5	62.73 ± 2.5	16.42 ± 0.2
	LiOH + CO_2_	168.37 ± 3	0.827 ± 0.002	85.29 ± 3	67.91 ± 2.3	1.84 ± 0.008
Al–Mg	25 °C					
	KOH	644.76 ± 5	1.514 ± 0.008	100.55 ± 4	113.45 ± 3	4.87 ± 0.01
	KOH + CO_2_	0.631 ± 0.005	0.671 ± 0.003	52.47 ± 2	49.95 ± 2	0.005 ± 0.001
	LiOH	104.06 ± 3	1.391 ± 0.007	44.44 ± 2	54.63 ± 2.4	0.79 ± 0.01
	LiOH + CO_2_	0.265 ± 0.002	0.165 ± 0.001	82.47 ± 3	55.64 ± 2.3	0.002 ± 0
	35 °C					
	KOH	1065.86 ± 5	1.521 ± 0.008	109.28 ± 5	116.51 ± 4	8.06 ± 0.03
	LiOH	212.09 ± 3	1.398 ± 0.007	42.88 ± 2	47.75 ± 2	1.60 ± 0.02
	45 °C					
	KOH	1961.87 ± 6	1.532 ± 0.006	121.50 ± 5	143.61 ± 4	14.83 ± 0.067
	LiOH	367.32 ± 3	1.404 ± 0.005	41.41 ± 2	45.76 ± 2	2.78 ± 0.05
	50 °C					
	KOH	2454.71 ± 7	1.536 ± 0.005	130.40 ± 5	158.18 ± 4	18.56 ± 0.07
	LiOH	522.12 ± 4	1.413 ± 0.004	39.60 ± 1	44.41 ± 2	3.95 ± 0.05

Changing the temperature from 25 to 50 °C affected the anodic and cathodic potentiodynamic polarization method of the Al–Mg anode in an electrolyte containing 1 M of both KOH and LiOH at a 1 mV/s voltage ramp rate. This experiment has been done, and the findings are exhibited in Fig. 3c,d. The potential of the Tafel zone was used to precisely predict many crucial variables of the electrochemical corrosion reaction. One of these essential variables *i*_corr._ for Al–Mg (in the two basic electrolytes) was accurately measured at various temperatures (25–50 °C). It was found that the temperature has essentially little influence on the electrochemical polarization graphs’ shape for the anodic and cathodic sides. On the other hand, when the temperature rises, there is a discernible movement towards lines with larger current densities^40^. By utilizing LiOH electrolyte, a minor change can be detected towards greater corrosion current density values of the cathodic arm at temperatures ranging from 25 to 50 °C. In contrast, when utilizing the KOH electrolyte, the corrosion current increases significantly at a temperature range at the same investigated temperatures. Besides, in both alkaline solutions, the corrosion voltage (*E*_corr._) is directed to the negative, increasing the temperature from 25 to 50 °C. But this shifting becomes higher in KOH solution compared to that of lithium hydroxide. The lithium-ion impact mainly raises the hydrogen overpotential of the alloy’s surface, which is responsible for this behavior by making the surface less reactive. That is to say, at the temperatures in concern, a significant overpotential is needed for hydrogen evolution on the alloy’s surface (25–50 °C). As a result, around the same temperatures, LiOH electrolyte results in less alloyed Al dissolving than KOH electrolyte. Consequently, the hydrogen overvoltage will diminish as the temperature (at the two basic electrolytes) increases. As a consequence, there is a rise in the hydrogen process of evolution, leading to a more significant current density. The slope values of the anode and cathode branches were calculated from Tafel plots (Table 3). The findings demonstrate that both the anodic (*β*_a_) and cathodic (*β*_c_) slopes (at 25 °C) are less when Li-ions are present compared to when KOH is present. Using LiOH as an electrolyte instead of KOH decreases the activity of the anodic and cathodic centers of the Al–Mg electrode. It has been noticed that the values of the Tafel slopes (*β*_a_ and *β*_c_) for the examined alloy diminish as the temperature increases (in the case of LiOH). This result provides more evidence for the hypothesis that the corrosion rate rises with rising temperature, as seen in Table 3. Conversely, using KOH, Tafel slopes (*β*_a_ and *β*_c_) increase with the temperature increase. This is because the anodic and cathodic arms of the polarization plots are becoming more active. This makes the anodic (*β*_a_) and cathodic (*β*_c_) slopes get greater. Moreover, in both solutions, the cathodic Tafel slope (*β*_c_) was higher than the anodic Tafel slope (*β*_a_) for the identical specimen. These findings may be clarified by the reality that the density of the cathodic current is smaller than its anodic one. Corrosion kinetics of Al–Mg in 1 M solutions of KOH and LiOH are determined to be cathodically controlled^41^.

The corrosion current density, denoted by *i*_corr._, is proportional to the average corrosion rate, denoted by *U*_corr._ (mm/y), which may be determined with the help of the formula shown below^42,43^:

4 $$ U_{Corr} \left( {mm/y} \right) = \frac{{3270 \times M \times i_{{{\text{corr}}.}} }}{d} $$

where the density of the corrosion current is denoted by *i*_corr._, and expressed in A cm^2^, *d* is the density of the material being corroded in g cm^3^, and *M* is the equivalent weight of the corroded material. The values of the corrosion rate of the Al–Mg electrode at each KOH and LiOH solution at various temperatures are listed in Table 3. The rate of corrosion for the electrode in the potassium hydroxide solution is higher than in the lithium hydroxide solution, which agrees with the corrosion current values. Likewise, the corrosion rate speeds up with a rise in the temperature; nevertheless, the acceleration of corrosion caused by a solution of lithium hydroxide is relatively mild compared to that caused by a solution of potassium hydroxide. This confirms that using lithium hydroxide solution instead of potassium hydroxide solution as an electrolyte of the Al–Mg alloy as an anode in alkaline batteries reduces electrochemical corrosion. The mechanism of reactions can be suggested in the absence of CO_2_ as the following:

In KOH solution:

5 $$ {\text{3Al }} + {\text{ 6OH}}^{ - } \to {\text{Al}}\left( {{\text{OH}}} \right)_{{3}} + {\text{Al}}_{{2}} {\text{O}}_{{3}} + {\text{ 3H}}^{ + } + {\text{ 9e}} $$

6 $$ {\text{2Mg }} + {\text{ 4OH}}^{ - } \to {\text{MgO }} + {\text{ Mg}}\left( {{\text{OH}}} \right)_{{2}} + {\text{ 2H}}_{{2}} {\text{O }} + {\text{ 4e}} $$

In LiOH solution:

7 $$ {\text{3Al }} + {\text{ 6OH}}^{ - } \to {\text{Al}}\left( {{\text{OH}}} \right)_{{3}} + {\text{ Al}}_{{2}} {\text{O}}_{{3}} + {\text{ 3H}}^{ + } + {\text{ 9e}} $$

8 $$ {\text{Mg }} + {\text{ 2OH}}^{ - } \to {\text{MgO}} + {\text{H}}_{{2}} {\text{O }} + {\text{ 2e}} $$

9 $$ {\text{2Li}}^{ + } + {\text{ 2OH}}^{ - } \to {\text{Li}}_{{2}} {\text{O }} + {\text{ H}}_{{2}} {\text{O}} $$

This expectation will be discussed in detail later by analysis of XRD data.

Comparing the densities of current of Tafel graphs in the presence of CO_2_ to those in its absence for Al–Mg electrode, Fig. 3b demonstrates that the current densities of Tafel graphs of anodic and cathodic branches are drastically reduced to extremely low values (almost from zero current densities). In addition, the corrosion potential (*E*_corr._) values are sharply changed from more negative (− 1.514 and − 1.391 V in the case of KOH and LiOH, respectively) to a very less negative direction (− 0.631 and − 0.265 V in the same two examined solutions containing CO_2_, respectively).

On the other hand, it’s observed that the change difference in *E*_corr._ as a result of the presence of CO_2_ in LiOH, is larger than in the KOH solution containing CO_2_ for Al–Mg alloy (see Fig. 3b). These results revealed the critical function CO_2_ plays in suppressing the corrosion process on the investigated electrode surface in the alkaline aqueous solutions. This pattern may be explained by the alloying components eventually creating an insoluble carbonate compound, such as Mg or Al, on the surface, which will be discussed later (using XRD). Therefore, the reaction between the surface and OH^−^ ions in the KOH solution containing CO_2_ may take place as follows:

10 $$ {\text{Al }} + {\text{ 3OH}}^{ - } \to {\text{Al}}\left( {{\text{OH}}} \right)_{{3}} + {\text{ 3e}} $$

11 $$ {\text{Mg }} + {\text{ CO}}_{{2}} + {\text{ 2OH}}^{ - } \to {\text{MgCO}}_{{3}} + {\text{ H}}_{{2}} {\text{O }} + {\text{ 2e}} $$

However, the suggested reaction in LiOH containing CO_2_ can be as follows:

12 $$ {\text{2Li}}^{ + } + {\text{ CO}}_{{2}} + {\text{ 2OH}}^{ - } \to {\text{Li}}_{{2}} {\text{CO}}_{{3}} + {\text{ H}}_{{2}} {\text{O}} $$

It is noticed that Al(OH)_3_ and MgCO_3_ are formed in KOH containing CO_2_, while Li_2_CO_3_ is formed only on the electrode surface in LiOH under the same conditions^44^. At the same time, H^+^ ions are released as follows:

13 $$ {\text{CO}}_{{2}} + {\text{ OH}}^{ - } \to {\text{CO}}_{{3}}^{{{2} - }} + {\text{ H}}^{ + } $$

So, the pH value of the examined alkaline solutions is significantly lowered from 13 to 9 experimentally. This means that the decrease in pH value influences protecting the surface from corrosion processes^45^.

It is important to note that when CO_2_ is present in alkaline solutions, the corrosion current and corrosion rate of the studied electrode drop very low^32^. Even though the data found are in direct opposition to the data that had been previously published in an acidic and neutral electrolyte containing CO_2_, the fact remains that a higher corrosion current and, thus, a higher corrosion rate result from the presence of CO_2_^27^. So, the present results can be a novelty in alkaline batteries' electrochemical efficiency. The lowest corrosion rate means that the use of Al as an electrode for discharge in alkaline battery applications is the highest; accordingly, the discharge capacity is the most significant^20^. It is possible to state that when the corrosion process is fully stopped, the capacity of aluminum as an anode rises to its theoretical value (2.98 h/g).

The corrosion parameters (such as *i*_corr._, *E*_corr._, etc.) at different temperatures for the Al–Mg and Al electrodes in the two investigated solutions are estimated and tabulated in Table 3. In the case of Al–Mg electrode the *i*_corr._ in the LiOH solution is much lower than that of the KOH solution at the investigated temperatures (25, 35, 45, and 50 °C). This is clear from the rate of corrosion, which decreases considerably with the utilization of lithium hydroxide as an electrolyte and Al–Mg as an electrode. In addition, magnesium atoms alloyed with aluminum play an important role. These magnesium atoms appeared distributed mostly at the borders of the steps^46^. As a result, the degradation of crystalline structures took place gradually along with the process. Hence, forming solid particles throughout the processes may block active sites during the aluminum dissolving reaction^19^. This may be seen in the images obtained from the SEM of the synthesized alloy. In addition, the quantity of corrosion output obtained by the LiOH electrolyte is lower than that produced by the KOH electrolyte of the identical anode, as seen in the XRD data (Fig. 6a,b), and the particles are believed to be more adherent to the surface. Consequently, the notable consequence of inhibiting hydrogen generation by magnesium alloying is that the surface aluminum particles are shielded by magnesium atoms, which leads to a substantial decrease in the dissolving of aluminum^47^. This means that the surface of the investigated electrode is more resistant to corrosion in a LiOH solution than in a KOH solution because of the generation of Li_2_O on its surface^48^. Thus, using this fabricated Al–Mg alloy as an anode and LiOH as an electrolyte was considered advantageous for alkaline batteries' durability and long life.

##### Thermodynamic parameters measurements

**Figure 4:**
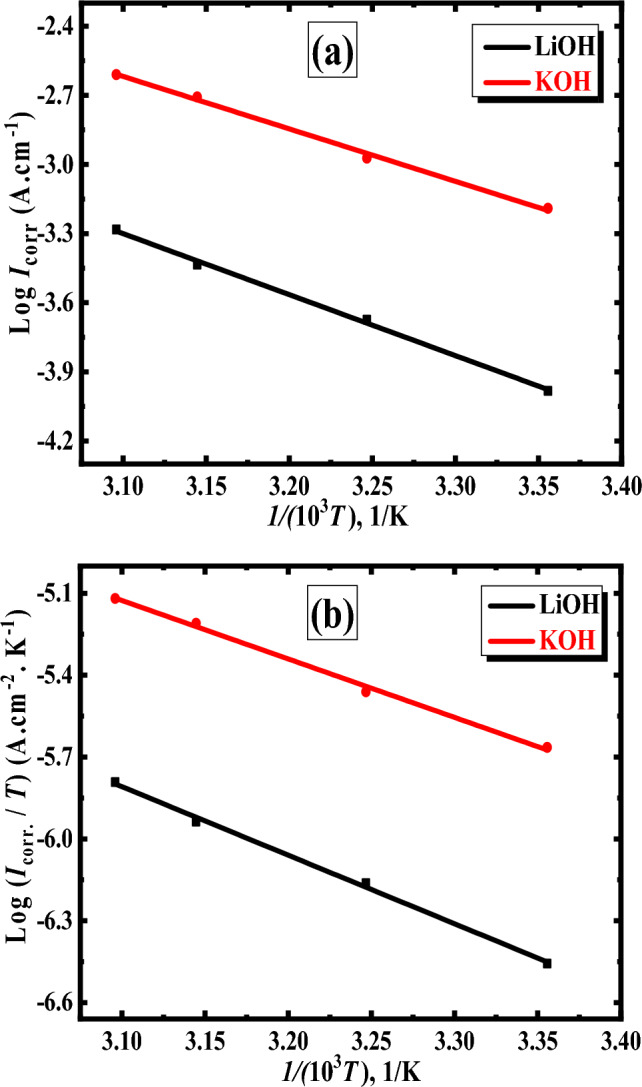
Arrhenius (**a**) and Eyring plots (**b**) for Al–Mg in 1 mol/L solution of both LiOH & KOH and at a temperature range of 25 to 50* °C*.

Using the Arrhenius equation allowed for the determination of the activation energy for electrochemical corrosion of the Al–Mg anode in both the KOH and LiOH test electrolytes^49^:

14 $${\text{log}}{i}_{corr.}={\text{log}}A-\frac{{E}_{{\text{a}}}}{2.303R}\frac{1}{T} $$

where *T* is the temperature in absolute terms measured in Kelvin, *A* is the frequency factor, *R* is the general gas constant in J/(mol. K), and *E*_a_ is the observed activation energy in kJ/mol. Figure 4a refers to the curves of the Arrhenius plots log (*i*_corr._) versus 1/*T* for the Al–Mg alloy in the two studied alkaline solutions. This plot displays straight lines of slopes − *E*_a_/2.303*R* and log *A* intercepts. The *E*_a_ values of the Al–Mg electrode in 1 molar of LiOH and KOH electrolytes have been precisely determined by using the slope of the presented lines of log (*i*_corr._) versus 1/*T*, as illustrated in Table 4. According to the findings, the *E*_a_ value of the Al–Mg anode in 1 M LiOH solution is more significant than that of the same anode in 1 M KOH solution. This mindset may be applied to the generation of Li_2_O, which suppresses the corrosion process and increases the activation energy barrier^39,41^. The second electrolyte, LiOH, has fewer active sites due to the adsorption of Li^+^ ions, which may cause a rise in the *E*_a_ values for this electrolyte. As a result, there is a reduction in the proportion of the anodic area to the cathodic area^50^. Alternatively, the existence of the Al_2_MgO_4_ phase in the investigated anode reduces and suppresses the hydrogen evolution process^51^. It has been shown that the presence of this phase throughout the fabricated electrode offers the optimal level of protection while maintaining the lowest possible corrosion rate. This attitude was validated by the estimated *E*_a_ value for the alloyed anode’s electrochemical corrosion rate in the two different solutions (KOH & LiOH). Nevertheless, lithium hydroxide solution is preferable to potassium hydroxide solution because it includes lithium-ion, which shields the anode and decreases corrosion by a larger proportion than in the first electrolyte. This makes lithium hydroxide solution a better electrolyte than KOH.

**Table 4:** EIS for the Al–Mg alloy in 1 M solution of both KOH & LiOH with and without CO_2_ at 25 and 50 °C.

T (°C)	Electrolyte (1 mol/L)	Parameters				
		*R*_s_ (ohm cm^2^)	*R*_p_ (ohm cm^2^)	*R*_ct_ (ohm cm^2^)	*CPE*	
					Y0 × 10 ^−5^	*n*
25	KOH	9.59 ± 0.02	11.7 ± 0.03	2.11 ± 0.01	5.28 ± 0.01	1 ± 0
25	LiOH	20.83 ± 0.05	424.77 ± 1.2	403.94 ± 0.9	7.68 ± 0.02	0.65 ± 0.002
25	KOH + CO_2_	12.93 ± 0.03	24,618.66 ± 8	24,605.73 ± 8	0.26 ± 0.001	0.98 ± 0.001
25	LiOH + CO_2_	40.75 ± 0.06	202,306.13 ± 19	202,265.38 ± 19	0.27 ± 0.001	0.88 ± 0.001
50	KOH + CO_2_	13.56 ± 0.02	7447.46 ± 3	7433.9 ± 3	0.53 ± 0.002	0.90 ± 0.001
50	LiOH + CO_2_	27.8 ± 0.05	16,496.09 ± 4	16,468.29 ± 4	0.62 ± 0.002	0.88 ± 0.001

In light of the findings, the activation energy (*E*_a_) value for the examined anode was greater (44.46 and 50.74 k joule/mol in KOH and LiOH, respectively) than twenty k Joule/mol in both evaluated solutions. This demonstrated that the surface reactions govern the corrosion processes that take place in the substrate^52,53^. Furthermore, the activation energy is lower in KOH compared with LiOH. This might indicate that corrosion occurs more in KOH solution than in LiOH.

As depicted in Fig. 4b, the plots represent the relationship between log (*I*_corr._ /*T*) and 1/*T* for the Al–Mg alloy explored in the two examined alkaline solutions. Two straight lines with varying slopes were shown using the Eyring Equation, and the intercepts have been determined from the equation as follows^54,55^:

15 $$ I_{{{\text{corr}}.}} = RT/Nh{\text{exp }}\left( {\Delta S/R} \right){\text{ exp }}\left( { - \Delta H/RT} \right) $$

where *h* stands for the constant of Planck, *N* for the number of Avogadro, Δ*H* for activation enthalpy, and Δ*S* for activation entropy.

In addition, the determined values for Δ*H* of the examined Al–Mg alloy in 1 M electrolyte of both KOH and LiOH are found to be + 42.17 and + 48.44, k joule/mol, respectively. At the same time, the values of Δ*S* are − 168.55 and − 159.64 J/(mol K), respectively. It was noticed that Δ*H* is positive and Δ*S* is negative. These conditions describe the corrosion process as an endothermic involving decreases in system entropy^56^. Furthermore, Δ*S* is large, proving the association process (the rate-determining). On the other hand, the Δ*S* value is low (in the case of LiOH solution), implying that the dissolution of Al–Mg was difficilitated compared to potassium hydroxide solution (the dissolution of Al–Mg was facilitated)^57^. Furthermore, this means the lithium hydroxide solution is less entropy than the potassium hydroxide solution. The higher value of Δ*H* with LiOH solution than that of the KOH solution of the examined alloy anode demonstrated that the solvation of Al is slow in the case of the LiOH electrolyte.

#### Measurements using Electrochemical Impedance Spectroscopy (EIS)

**Figure 5:**
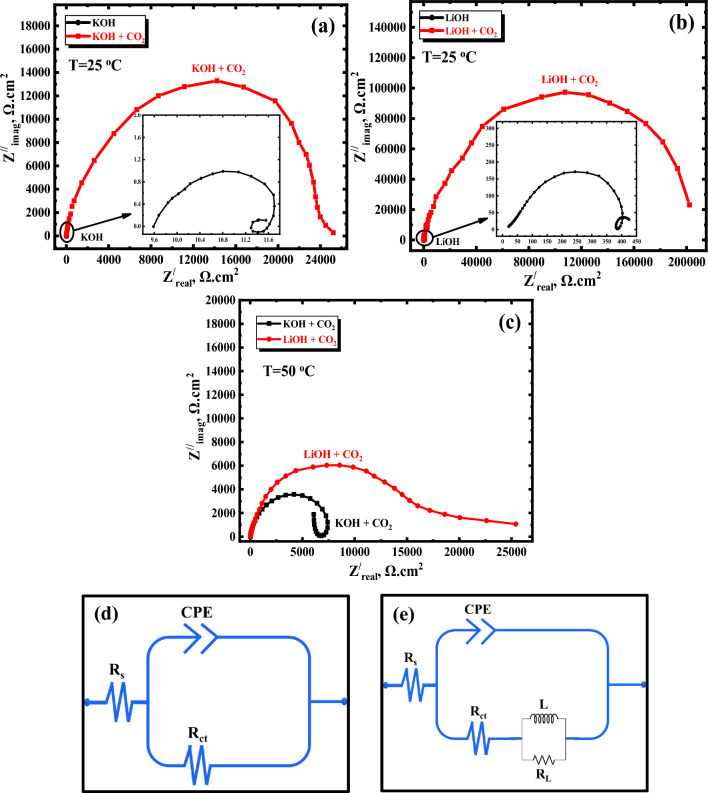
Nyquist plots for Al–Mg in 1 M solution of KOH and LiOH with and without CO_2_ at 25 °C (**a**,**b**), and 50 °C (**c**) which were accomplished vs. open circuit potential, AC voltage 10 mV, and frequencies range of 10,000–1 Hz. (**d**) and (**e**) represent the equivalent circuits.

Figure 5a,b displays the EIS Nyquist plot for the Al–Mg electrode in 1 M of KOH and LiOH, respectively with and without CO_2_ (at the *E*_corr._) at 25 °C. The results acquired from the potentiodynamic polarization method were analyzed, and the EIS of the studied anode was looked at to substantiate those findings. EIS data are listed in Table 4 for Al–Mg alloy in both alkaline electrolytes in the presence and absence of CO_2_ at 25 °C. Table 4 lists significant characteristics that were derived from a Nyquist plot, including the element of constant phase (*CPE*), the resistance of the solution (*R*_s_), the polarization resistance (*R*_p_), and the resistance of the charge transfer (*R*_ct_).

The diameter of the semicircle for the EIS plot for the examined anode in the LiOH electrolyte is much larger than the diameter of the identical alloy in the other alkaline one (KOH), as observed in Fig. 5a,b. This demonstrates that the impedance becomes more capacitive in the LiOH solution. This illustrates that the Li^+^- ion content was significantly the protection against corrosion. The findings exhibit that the Al–Mg anode’s corrosion resistance in LiOH solution is superior to that of KOH. On the other hand, Fig. 5a,b, which represents the presence of carbon dioxide with the two alkaline solutions of the investigated alloy, finds that the radius of the semi-circle is very large compared to that in the absence of carbon dioxide, and this shows the extent of the great resistance to corrosion process in the mentioned solution with CO_2_. This is clear from the charge transfer values, which are 24,605.73 and 202,265.38 Ω cm^2^ for the KOH and LiOH, respectively in the presence of CO_2_ for Al–Mg alloy (listed in Table 4). This also verifies that these data are in excellent agreement with Tafel polarization graphs. Figure 5c displays the EIS Nyquist plot for the investigated electrode in 1 M of KOH and LiOH with CO_2_ (at the *E*_corr._) at 50 °C. As it is clear from the curves and the calculations in Table 4, the resistance decreases with increasing temperature, and this is completely consistent with the Tafel results.

**Figure 6:**
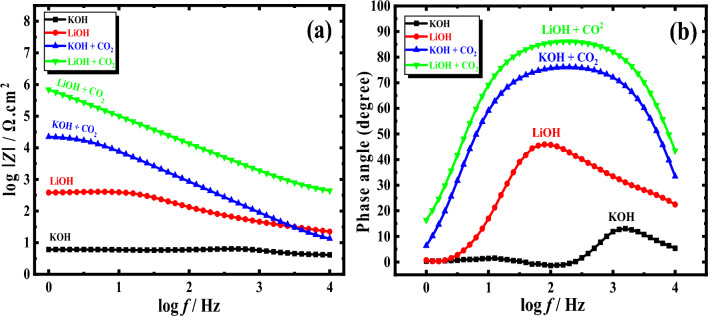
Bode plots (**a**), and phase (**b**) for Al–Mg in 1 M solution of KOH and LiOH with and without CO_2_ at 25 °C, which were accomplished vs. open circuit potential, AC voltage 10 mV, and frequencies range of 10,000–1 Hz.

The implemented design of the corresponding circuit for fitting the received results is depicted in Fig. 5d,e, where (d) represents the equivalent circuit for a Nyquist semi-circle and (e) for a Nyquist semi-circle with an inductive loop. Figure 6a,b shows the EIS results in Bode and phase plots. These charts demonstrate without a reasonable doubt the existence of two-time constants. According to the modulus vs. log frequency graphs, the absolute impedance values for the investigated alloy in the LiOH solution are significantly more than those in the KOH solution (especially with CO_2_).

For modeling purposes, such as the roughness of a coating layer or the non-ideality of the electrode surface, the constant phase element (*CPE*) is frequently utilized in place of a capacitor (*C*_dl_). The typical parameters of the *CPE* are Y0 and *n*, which are illustrated in Table 4. As a result, in many real-world situations, a *CPE* is employed in place of a capacitor when analyzing the electrochemical impedance spectra^58^. A category of characteristics related to both the surface and the electroactive components is referred to as *CPE*. Because of the dispersion of relaxation times brought on by inhomogeneities such as adsorption, diffusion, and surface roughness/porosity, the *CPE* is essential^59^. Additionally, the *CPE* has an exponent called “n” that is used to look at changes in the metal/solution interface. The near-unity values of *n*^59^, which show the predominant capacitive response as in the current investigation, are caused by the frequency dispersion created by an appropriately dispersed current on the surface of the electrode.

In light of these findings, it may be deduced that the corrosion protection offered by the LiOH solution for the Al–Mg alloy was superior to that offered by the KOH solution (especially with CO_2_). Figure 5a,b provides the experiment results conducted under low-frequency settings, during which an inductive capacitance loop was seen (without using CO_2_). This phenomenon was attributed to generating an oxide film on the Al–Mg anode surface, illustrated in this figure. This suggests that the surface of the investigated anode is coated with an oxide film throughout pretreatment in the evaluated solution prior to each experiment. This feature may be demonstrated by the simple interaction of the metal alloy with oxygen to generate the surface protective coating. It could be related to the dielectric characteristics of the layer’s surface. However, the reason for the huge inductive loop remained unknown at lower frequencies. It is conceivable to hypothesize that this propensity is caused by the ions migrating over the alloy’s surface due to physical phenomena. This anticipation may become more prominent when an inductive loop incorporates the adsorption of intermediates that charge through its structure. At the contact between the metal and the oxide, one may anticipate the formation of OH^−^ or O^2−^ ions^20^. However, in the case of LiOH, the inductive loop is completely formed compared with that of KOH due to the adsorption of Li^+^ ions on the surface.

### Characterization of the produced compounds after corrosion on the anode surface in both electrolytes (KOH and LiOH)

#### XRD technique

**Figure 7:**
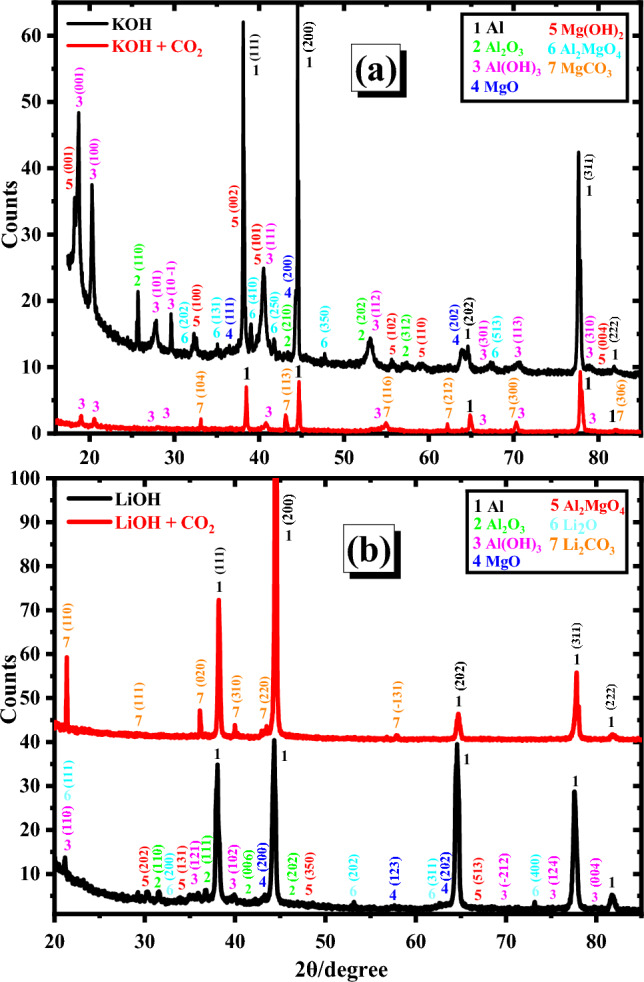
Patterns of XRD for the electrochemically synthesized oxide films on the Al–Mg anode in 1 M of both KOH (**a**) and LiOH (**b**) without and with CO_2_ at the active region.

Figure 7 presented the XRD charts of the corrosion product formation on the examined electrode surface (after the Tafel polarization technique) in 1 M of both KOH (a) and LiOH (b) solutions at ambient temperature at the active area. Some differences are noticed by examining the XRD results shown in Figs. 1 and 7 without CO_2_. New peaks for Al_2_O_3_, Al(OH)_3_, MgO, and Mg(OH)_2_ are displayed in Fig. 7a. These peaks are absent in the case of a surface composed entirely of pure Al–Mg (Fig. 1). The peak intensities for Al and Al_2_MgO_4_ phases are lower than those of the pristine surface. The pattern of XRD for the oxide film that forms on the surface of the anode in the case of a LiOH solution and also in the absence of CO_2_ is shown in Fig. 7b. Also, some new peaks appeared for Al_2_O_3_, Al(OH)_3_, MgO, and Li_2_O compared to the pristine surface. Besides, Al_2_O_3_, MgO, and Al(OH)_3_ showed greater peak intensities for the investigated electrode in KOH than those in the LiOH solution. XRD patterns of the Al–Mg alloy in LiOH electrolyte exhibit no peaks for Mg(OH)_2_. Besides, the intensities of Al and Al_2_MgO_4_ peaks in the case of KOH as an electrolyte are lower than those in LiOH of the same electrode. This demonstrated that a significant portion of the alloy surface was coated with Al_2_O_3_ & Al(OH)_3_, but a minor coated with MgO & Mg(OH)_2_ in the KOH solution was observed. Conversely, in the LiOH solution, the surface of the alloy was majorly coated with Li_2_O, but minor coated with Al_2_O_3_, Al(OH)_3_, and MgO was observed. Moreover, XRD, SEM, and EDX data confirmed that the quantity of oxide and hydroxide of aluminum on the Al–Mg electrode in the KOH electrolyte is higher. A large portion of the surface is coated with Al_2_O_3_ in addition to MgO & Mg(OH)_2_. While utilizing LiOH, most of the surface of the Al–Mg electrode is coated with lithium oxide in addition to low percentages of Al_2_O_3_, Al(OH)_3_, and MgO.

In contrast, Al(OH)_3_ and MgCO_3_ were solely formed on the electrode surface, according to XRD measurements (Fig. 7a), in KOH containing carbon dioxide. This suggests that the existence of carbon dioxide in KOH can stop the creation of Al_2_O_3_ and MgO with the disappearance of the Al_2_MgO_4_ phase on the surface, produced in non-additive KOH media. This suggests that the inhibiting effect of the corrosion process results from the development of insoluble MgCO_3_ and Al(OH)_3_ on the surface in the existence of carbon dioxide. XRD measurements in LiOH with CO_2_ are shown in Fig. 7b. It is noted that just the electrode surface produces significant amounts of Li_2_CO_3_.

By comparing the results of XRD in Fig. 7b of LiOH electrolyte without and with CO_2_, the formation of Al_2_O_3_, Al(OH)_3_, MgO, and Li_2_O phases in addition to the disappearance of Al_2_MgO_4_ phase occurs in the absence of CO_2_. This demonstrates that with CO_2_, the active sites on the surface are considerably inhibited by the production of Li_2_CO_3_ exclusively on the surface. As a result, the highest decrease in the *i*_corr._ value and the largest change to a less negative direction of the *E*_corr._ (Table 3) in LiOH containing CO_2_ might be attributed to the creation of insoluble Li_2_CO_3_. Therefore, conclusions drawn from Tafel plots support the data from XRD.

The following cards were used for detection and matching the produced materials on the corroded electrodes; COD#4,313,210 (Al), COD#1,010,951 (Al_2_O_3_), COD#1,536,398 (Al(OH)_3_), COD#9,013,223 (MgO), COD#1,010,484 (Mg(OH)_2_), COD#5,000,120 (Al_2_MgO_4_), COD#9,000,096 (MgCO_3_), COD#1,011,195 (Li_2_O), and COD#9,009,642 (Li_2_CO_3_).

##### SEM with EDX analysis

**Figure 8:**
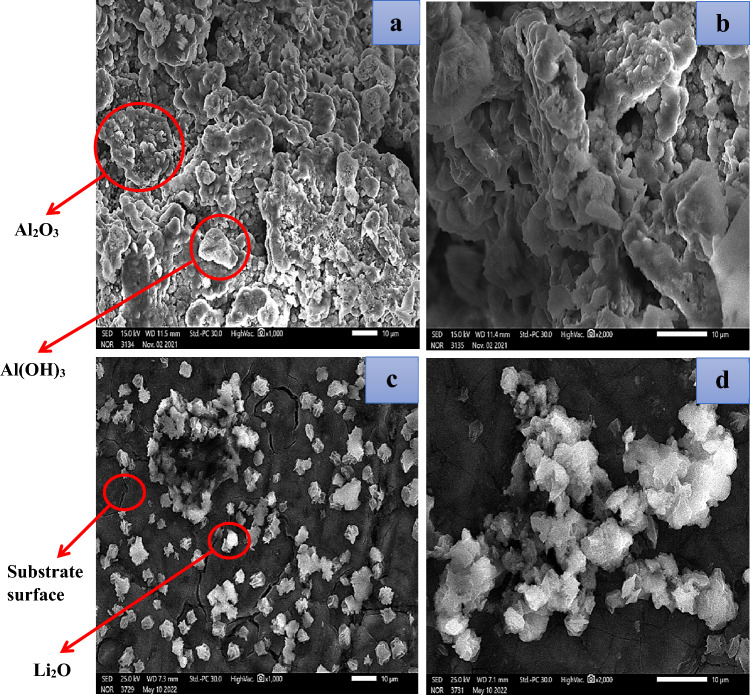
Captured images from SEM of the Al–Mg alloy in KOH solution with a magnification of × 1000 (**a**) and × 2000 (**b**), in LiOH solution with a magnification of × 1000 (**c**), and × 2000 (**d**).

Figure 8 exhibits the images of SEM analysis of the corroded surface (after the Tafel polarization technique) of the Al–Mg anode in 1 M of both KOH (a & b) and LiOH (c & d) at room temperature at the activated area. Figure 8a,b demonstrates that a thick porous film mostly coats the surface of Al–Mg and is only weakly linked to the surface. The particle size of the coated film (corrosion product) is large, and the surface distortion is significant. The particles of the coated film have many different shapes, which can be returned to the generation of Al(OH)_3_, Mg(OH)_2_, MgO, and Al_2_O_3_ composites. Also, the percentage of Al_2_O_3_ & Al(OH)_3_ formed on the surface is high, which is clear from the high intensity of these compounds, as shown in the XRD technique (Fig. 7a).

Nevertheless, the scanning electron micrograph of the synthesized alloy in LiOH (Fig. 8c,d) reveals that the anode surface is mostly entirely coated by an adhered passivation layer. The particulates are fewer and more surface-adherent than in the KOH solution. This suggests that the interaction of LiOH as an electrolyte with the Al–Mg electrode reduces the electrochemical corrosion prevents the formation of Mg(OH)_2_, retards the generation of MgO compared with that in KOH, and stimulates the building up of Li_2_O. Furthermore, the percentage of Al_2_O_3_ & Al(OH)_3_ produced on the surface is low compared to that of another alkaline solution (KOH), which is clear from the intensity of these compounds, as shown in XRD. (Fig. 7b).

**Figure 9:**
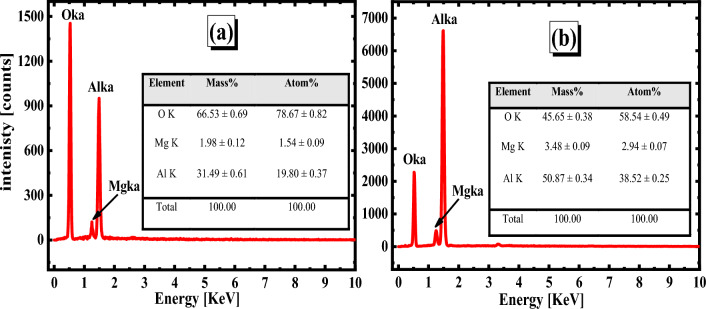
EDX analysis charts of the oxide layer formed on the Al–Mg surface in 1 M solution of each KOH (**a**) and LiOH (**b**) at the active region.

These results confirm the values of *i*_corr_ for the tested electrode in LiOH electrolyte are smaller than that of the same alloy in KOH. It is possible to deduce that SEM images of lithium hydroxide can be used as an electrolyte of the battery instead of potassium hydroxide. This demonstrates that Al–Mg alloy as an electrode provides greater protection due to the production of lithium oxide and hinders the formation of Mg(OH)_2_. Also, it minimizes the creation of Al_2_O_3_ & Al(OH)_3_. i.e., protect the Al and Mg in the alloy from the corrosion process. To ascertain the amount of oxides and elements produced in the active region in the surface layer, an EDX examination was carried out (after the Tafel polarization technique). EDX diagrams are displayed in Fig. 9a,b. In the case of the KOH electrolyte, as compared to the LiOH electrolyte, a coating is generated on the surface of Al–Mg alloy with a more significant concentration of oxygen. For LiOH and KOH electrolytes, the amount of Al detected in the produced oxide layer was 50.87% and 31.49%, respectively. As well as the percentage of Mg element is detected as 3.48 and 1.98% for LiOH and KOH solutions, respectively.

In contrast, the percentage of oxygen in the film is low (45.65%) of LiOH compared with that of KOH (66.53%). This pattern gave credence to the conclusion reached using the SEM. In this circumstance, the activated surface of the examined anode is decreased, and most of the surface is coated by lithium oxide. The situation occurs when the solution in concern is LiOH. In other words, the protection of the alloy against the electrochemical corrosion process increased.

**Figure 10:**
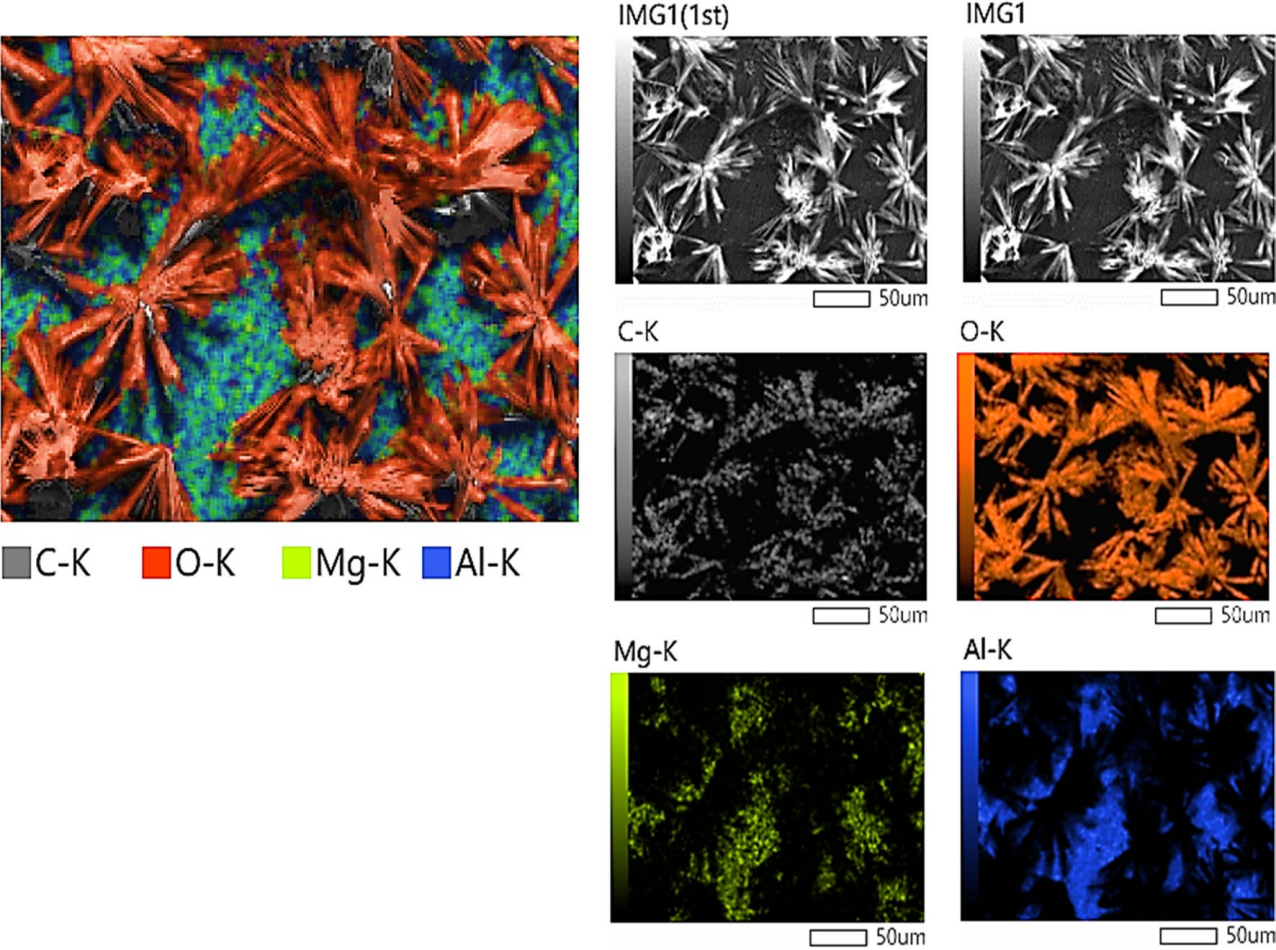
SEM mapping images of the layer electrochemically formed on Al–Mg alloy in 1 M LiOH solution containing saturated CO_2_ on the activated area at 25 °C.

Figure 10 exhibits the SEM mapping images for the electrochemically polarized surface of Al–Mg alloy in the solution of 1 M LiOH saturated with carbon dioxide at 25 °C. It is noted that the surface of the alloy is covered with two layers. The first has small particles of aluminum and magnesium (these are the original components of the alloy), and the second layer consists of large particles in the form of a like-star structure. This is due to the presence of carbon and oxygen as carbonates, as confirmed by XRD, distributed regularly on the surface of the alloy and highly bound to it. This is evidence for the presence of lithium carbonate only and the absence of aluminum or magnesium oxide (lithium is a light element that does not appear in SEM mapping). The previous results were confirmed by the X-ray analysis (Fig. 7).

### Charge–discharge technique

**Figure 11:**
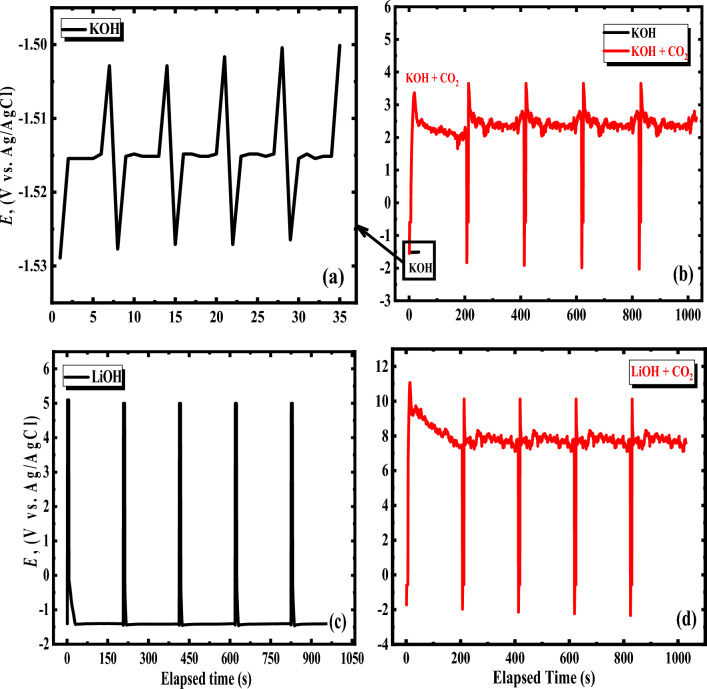
Charge–discharge of Al–Mg alloy in 1 M solution of both KOH (**a**) and LiOH (**c**), in addition to the presence of CO_2_ with KOH (**b**) and LiOH (**d**) at applied current density ± 1 mA cm^−2^, and 25 °C.

**Figure 12:**
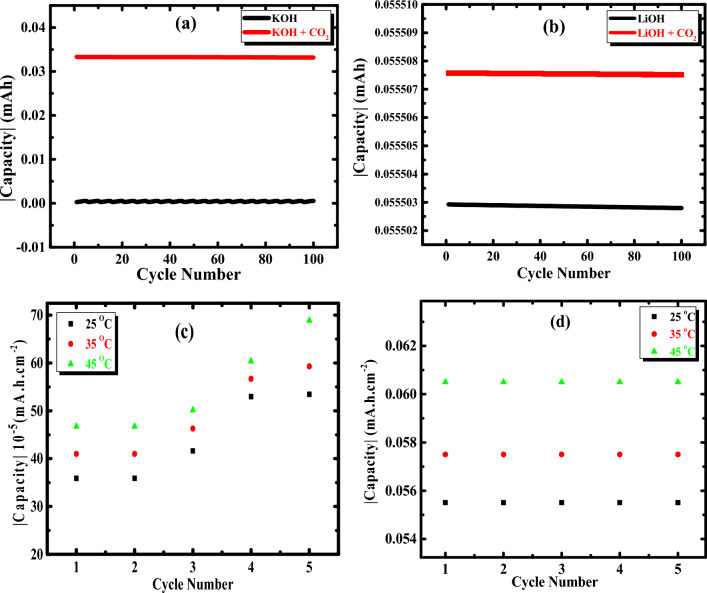
The alloyed anode capacitance in 1 M of KOH (**a**) and LiOH (**b**) in the absence and exitance of carbon dioxide in each of the two examined solutions at 25 °C for 100 cycles. The capacitance of the Al–Mg in 1 M solutions of both KOH (**c**) and LiOH (**d**) at various temperatures (25, 35, 45 Celsius) and density of applied constant current (± 1 m amp cm^−2^).

A galvanostatic charge–discharge method was carried out to investigate the real influence of Li-ion and saturated carbon dioxide on implementing the alkaline battery. Figure 11 exhibits the galvanostatic charging-discharging plots of the investigated Al–Mg alloy at a fixed current density of ± 1 m amp cm^−2^ in 1 M of the two examined solutions (KOH and LiOH) with and without saturated carbon dioxide. Notably, the discharging potential reaches plateaus of the alloy in LiOH solution are significantly higher than that of KOH electrolyte (without CO_2_), confirming its impedance behavior and lifetime are powerfully improved. The findings above provide conclusive evidence that the Al–Mg anode has a superior energy efficiency (when using LiOH solution) and has significantly been enhanced compared with the KOH solution. The cycle stability of the anode under consideration was investigated by utilizing a galvanostatic charge–discharge process, and a constant current density of 1 m amp/cm^2^ was applied throughout the experiment. Consequently, the electrolyte that is utilized in alkaline batteries has a significant impact on the overall energy predicted value. As such, to enhance the Al–Mg anode’s discharging effectiveness, the anode’s spontaneous self-discharge should be slowed down as much as conceivable. As displayed in Fig. 11a,c, the Al–Mg alloy exhibited a very long discharging time (200 s per cycle) and a very short charging time (5 s per cycle) in the second solution (LiOH). Conversely, the first solution (KOH) shows a very short discharge time with 1 s per cycle and a long charging time (6 s per cycle) without CO_2_. But, with CO_2_, the solution of KOH shows a very short charging time of 5 s per cycle and a long discharging time (200 s per cycle). Also, the charging-discharging potentials of the Al–Mg alloy in the two studied electrolytes significantly improved, as shown in Fig. 12b,d. This is a huge difference in the previous values between the absence and presence of saturated carbon dioxide^32^.

This means that the improvement of the Al–Mg anode utilizing lithium hydroxide (as an alkaline electrolyte instead of potassium hydroxide) with CO_2_ is suggested. The potential and time of the charge–discharge process for the investigated electrode in the two examined electrolytes are shown in Table 5.

**Table 5:** Time and Potential of Charging–Discharging process for the Al–Mg alloy at constant current densities ± 1 m amp cm^−2^ in 1 molar of each potassium and lithium hydroxide in the presence and absence of CO_2_ per cycle.

Solution (1 M)	Time of charging (s)	Time in discharging (s)	Charging potential (volts)	Discharging potential (volts)
KOH	6 ± 0.05	1 ± 0.01	− 1.50 ± 0.01	− 1.53 ± 0.01
KOH + CO_2_	5 ± 0.04	200 ± 1	3.69 ± 0.03	− 1.91 ± 0.02
LiOH	5 ± 0.04	200 ± 1	5.10 ± 0.04	− 1.45 ± 0.01
LiOH + CO_2_	5 ± 0.04	200 ± 1	10.2 ± 0.05	− 2.03 ± 0.02

Both the energy and the charge efficiencies of a battery are essential performance factors, and they may be determined using the formula as follows:

16 $$ {\text{Charge efficiency}}= \frac{{{\text{charge from discharging}}}}{{{\text{total charge consumed}}}} \times 100\% $$

17 $$ {\text{Energy efficiency}} = \frac{{{\text{energy from discharging}}}}{{{\text{total energy consumed}}}} \times 100{\text{\% }} $$

The energy and charge efficiencies for the Al–Mg alloy in the two alkaline media without and with CO_2_ are estimated and listed in Table 6. It can be observed that there is a vast difference in the charge and energy efficiency between the use of the two solutions, unlike potassium hydroxide, which has energy and charge efficiency values of 15.50 and 14.29%, respectively. The Li-ions appear to have high energy and charge efficiency (98.67 and 97.56%, respectively) without CO_2_. Conversely, CO_2_ in KOH results in a very high energy and charge efficiency (98.20 and 97.56%, respectively) until nearby, equaling the values of LiOH.

**Table 6:** Energy and charge efficiencies for Al–Mg alloy in 1 M of each KOH and LiOH with and without CO_2_ for the alkaline battery at constant applied current (± 1 mA cm^−2^).

Electrolyte (1 molar)	Charge efficiency (%)	Energy efficiency (%)
KOH	14.29	15.50
KOH + CO_2_	97.56	98.20
LiOH	97.56	98.67
LiOH + CO_2_	97.56	98.90

Based on the findings, utilizing LiOH instead of KOH solution has enhanced the alkaline Al–Mg battery’s efficiency. The capacitance versus cycle number curves for the Al–Mg alloy at constant applied current (± 1 m amp cm^−2^) in 1 M solutions of both KOH and LiOH with and without CO_2_ at ambient and different temperatures, as shown in Fig. 12a–d. It is clear from Fig. 12a that the discharging capacitance in the solution of KOH (without CO_2_) seems to be constant and relatively low during the discharging process. But, in the case of saturated CO_2_, the capacitance is very high and constant. This confirms the stability of the system.

On the other hand, using LiOH without CO_2_, the discharging capacitance is still constant and high during the discharge process compared with pure KOH. In comparison, CO_2_ enhanced the discharge capacitance and made it more negative, as shown in Fig. 12b. It can be concluded that, under all circumstances, the charge–discharge capacitance increases when using LiOH solution, especially in the presence of CO_2,_ than in KOH solution. Table 7 shows the capacitance for the Al–Mg alloy at constant current densities ± 1 m amp cm^−2^ in 1 molar of both potassium and lithium hydroxide with and without CO_2_ per cycle. From these results, it can be noticed that there is a large difference in the storage capacity when using lithium hydroxide (especially with CO_2_) compared with that of potassium hydroxide. This demonstrates that Li-ion has a significant impact on the suppression of self-discharge and an improvement of discharge efficiency via delaying Al–Mg dissolving.

**Table 7:** Capacitance for the Al–Mg alloy at fixed densities of the current (± 1 m amp cm^−2^) in 1 molar of both potassium and lithium hydroxide with and without carbon dioxide per cycle.

Tested solution (1 mol/L)	Capacitance of charging (mAh/cm^2^)	Capacitance of discharging (mAh/cm^2^)
Pure KOH	5.3 × 10^–4^	2.7 × 10^–4^
KOH with CO_2_	3.3 × 10^–2^	2.6 × 10^–4^
Pure LiOH	5.6 × 10^–2^	8.9 × 10^–4^
LiOH with CO_2_	5.6 × 10^–2^	3.9 × 10^–4^

By testing the effect of the temperature on each of the two solutions (KOH & LiOH) on the storage capacity of the examined electrode during the charging process, it was found that the charging capacitance of the Al–Mg electrode is slightly increased (i.e. the surface of the electrode undergoes a certain reaction which provides the electrochemical corrosion) with increasing the temperature from 25 to 45 °C in the KOH solution (as shown in Fig. 12c). But, in the LiOH solution, charging capacitances are nearly constant with increasing temperature (i.e. no reaction occurs), as shown in Fig. 12d. This confirms that the specific capacitance of the Al–Mg electrode increases by the utilization of the LiOH as an electrolyte instead of a KOH electrolyte.

Consequently, one may conclude that lithium ions have a considerable impact on exerting a positive effect on the efficiency of Al–Mg as an anode in an alkaline solution. As a result of these factors, the Al–Mg alloy develops the retardation of the corrosion attack, a longer discharge duration, and a larger capacitance. This behavior can be attributed to the influence of lithium ions on the mentioned system.

## Conclusion

The present work focuses on understanding the role of CO_2_ (in both the two studied electrolytes) on the electrochemical performance and the possibility of its use in alkaline batteries. In the course of this research, a comparison was established between the electrochemical characteristics of Al–Mg alloy as an anode for Al-air batteries in KOH and LiOH solutions, both in the absence and presence of CO_2_. The data exhibited a much lower corrosion current density (i_corr._) in the LiOH solution than in the KOH solution. This behavior is supported by the positive displacement of corrosion potential (*E*_corr._) in LiOH solution confronted with KOH. This indicates that Li-ion content influences minimizing the corrosion of Al–Mg alloy due to its adsorption on the surface. The evaluated data from both Tafel plots and electrochemical impedance spectroscopy (EIS) techniques exhibited very low corrosion rate values in the presence of CO_2_ of the two examined solutions compared with those in their absence. In addition, the corrosion potential (*E*_corr._) values are sharply changed from more negative values to a less negative direction in the two solutions containing CO_2_. However, the change difference in *E*_corr_ in LiOH is larger than in KOH solution under the same conditions. This means that the layer formed on the surface in LiOH containing CO_2_ is more adhering and has a higher protecting effect than that in KOH, also containing CO_2_. Analysis of the corroded surface utilized XRD, SEM, and EDX exhibit that Li_2_O is formed on the alloy surface in LiOH, which protects the alloy surface more than in KOH in the absence of CO_2_. However, the surface is considerably inhibited by the production of Li_2_CO_3_ in addition to the formation of Li_2_O in LiOH containing CO_2_. This exhibits more protection from the corrosion in LiOH containing CO_2_ compared with that in KOH. Tests of charge and discharge show that the potential has moved toward a high positive amount, in addition to the discharge time and capacitance increase with the utilization of LiOH electrolyte. It is observed that the specific capacitance of the Al–Mg alloy increases with the utilization of LiOH as an electrolyte instead of KOH. This demonstrates that Li-ion has a significant impact on the suppression of self-discharge and an improvement of discharge efficiency via delaying Al–Mg alloy dissolving. Consequently, one may conclude that lithium ions have a considerable impact on exerting a positive effect on the efficiency of Al–Mg alloy as an anode in alkaline batteries. As a result of these factors, the studied alloy develops a reduced corrosion attack, longer discharge duration, and a higher capacitance due to the presence of both Li-ion and CO_2_ in the electrolyte.

## Data Availability

The datasets used and/or analyzed during the current study are available from the corresponding author on reasonable request.
